# Aftercare of Childhood Cancer Survivors in Switzerland: Protocol for a Prospective Multicenter Observational Study

**DOI:** 10.2196/18898

**Published:** 2020-08-26

**Authors:** Sibylle Denzler, Maria Otth, Katrin Scheinemann

**Affiliations:** 1 Division of Oncology-Hematology Department of Pediatrics Kantonsspital Aarau Aarau Switzerland; 2 Institute of Social and Preventive Medicine University of Bern Bern Switzerland; 3 Division of Oncology-Hematology University Children’s Hospital Basel Basel Switzerland; 4 University of Basel Basel Switzerland; 5 Department of Pediatrics McMaster Children’s Hospital Hamilton, ON Canada; 6 McMaster University Hamilton, ON Canada

**Keywords:** childhood cancer survivors, long-term follow-up care, transition, Switzerland

## Abstract

**Background:**

Most children and adolescents diagnosed with cancer become long-term survivors. For most of them, regular follow-up examinations to detect and treat late effects are necessary, especially in adulthood. The transition from pediatric to adult-focused follow-up care is a critical moment for childhood cancer survivors (CCSs); a substantial proportion of CCSs are lost to follow-up in this transition process and do not attend follow-up care in adulthood. This can have serious effects on survivors’ health if late effects are not discovered in a timely fashion.

**Objective:**

In this study, we primarily assess the current follow-up situation, related needs, and knowledge of adolescent and young adult CCSs who have transitioned from pediatric to adult-focused follow-up care. As secondary objectives, we evaluate transition readiness, identify facilitating factors of transition and adherence to long-term follow-up (LTFU) care, and compare three different transition models.

**Methods:**

The Aftercare of Childhood Cancer Survivors (ACCS) Switzerland study is a prospective, multicenter, observational study that was approved by the ethics committee in February 2019. We are recruiting CCSs from three pediatric oncology centers and using questionnaires to answer the study questions.

**Results:**

To date, we have recruited 58 participants. The study is ongoing, and recruitment of participants will continue until January 2021.

**Conclusions:**

The ACCS study will provide information on CCSs’ preferences and expectations for follow-up care and their transition into the adult setting. The results will help improve the LTFU care and cancer knowledge of CCSs and subsequently enhance adherence to follow-up care and reduce loss to follow-up in adulthood.

**Trial Registration:**

ClinicalTrials.gov NCT04284189; https://clinicaltrials.gov/ct2/show/NCT04284189?id=NCT04284189

**International Registered Report Identifier (IRRID):**

PRR1-10.2196/18898

## Introduction

### Background

Most children and adolescents diagnosed with cancer become long-term survivors and need lifelong follow-up care [1]. Childhood cancer survivors (CCSs) are vulnerable to physical and psychosocial chronic medical conditions, so-called late effects. Some late effects are characteristic of CCSs, while most of them are also associated with aging in the general population but develop earlier in CCSs. Three out of four 5-year CCSs treated between 1966 and 1996 with a median follow-up time of 17 years experienced at least one late effect, 37% of which may be life threatening [2]. The goal of follow-up care is to detect late effects early and to intervene, treat, or slow their progression. Finally, follow-up care aims to reduce the burden of late effects and improve CCSs’ quality of life. Studies show that long-term follow-up (LTFU) care improves the detection of late effects and survivors’ health behavior and knowledge, encourages health care use, and decreases survivors’ distress levels [3].

As CCSs reach adulthood, questions and issues arise that pediatric oncologists are no longer expert in addressing and, therefore, adult physicians are needed. This process of change in health and follow-up care is known as transition. Transition has been described as the planned movement of adolescent and young adult (AYA) patients with chronic health conditions from child-centered to adult-oriented health care systems. The preparation of adolescents for transition should start several years before the actual transition occurs. Adolescents need to acquire the knowledge and skills to assume independent responsibility for their health care in the adult system. The process of transition ideally should address medical, psychosocial, and vocational needs. During transition, care shifts from family-centered pediatric to independent patient-centered health care as survivors enter adulthood. The transition of CCSs is a critical moment in LTFU care that may affect survivors’ LTFU clinic attendance and may cause loss to follow-up [4-7]. There is a clear and steady decrease in the proportion of CCSs who attend a clinic visit as time since treatment completion increases [4]. This is particularly concerning since CCSs are at greater risk of late effects as time passes [8,9]. One reason for loss to follow-up seems to be CSSs’ lack of knowledge regarding their diagnosis, treatment, and risk of developing late effects [10,11]. This factor can be influenced by educating CCSs. Another reason is the lack of knowledge about late effects and the need for further follow-up examinations with adult physicians who should provide this care [12].

To date, it is not clear which transition and LTFU care model is the best based on the existing health care system. Many different models of LTFU care exist: (1) models calling for transition to primary care physicians, (2) shared-care models with LTFU care provided by the primary care physician in collaboration with the oncology team, (3) models suggesting transition from pediatric to adult oncologists, or (4) models proposing transition to specialized LTFU clinics, which provide LTFU care in multidisciplinary teams [13,14]. In addition to the debate surrounding the model of LTFU care, there is debate on the right time point of transition. Some study groups have developed and used scores to assess the transition readiness of AYA patients with chronic diseases, such as inflammatory bowel disease [15], cystic fibrosis [16], or congenital heart disease [17]. Klassen et al developed tools to assess CCSs’ readiness for transition [18], and Schwartz et al are currently developing a Transition Readiness Inventory Item Pool, which measures socioecological components relevant for successful transition [19]. However, evidence collected prospectively during the transition process addressing the needs of young adult CCSs during their transition into adult LTFU care in different settings is limited.

Nine pediatric oncology centers treat children and adolescents up to the age of 18 years diagnosed with cancer in Switzerland. Like the initial treatment, follow-up care is provided in these nine centers until at least the age of 18 years and usually for longer. A recent survey including the division head or the responsible staff physician for follow-up care of each of the nine centers showed differences in follow-up care between centers, especially concerning transition [20]. These differences were also noted in the current position statement from the Pediatric Swiss Long-Term Follow-Up Working Group [21]. This working group aims to achieve harmonization between the centers. Both publications reflect the current situation of physicians in Switzerland, but the needs of Swiss CCSs are still unknown.

### Objectives

The primary objectives of the Aftercare of Childhood Cancer Survivors (ACCS) Switzerland study are to assess the current follow-up situation, related needs, and knowledge of AYA survivors of childhood cancer undergoing LTFU care transitions from pediatric to adult-focused follow-up care. By including three pediatric oncology centers with different transition and LTFU care models, we aim to investigate which model or which parts of the models have the best fit for Swiss CCSs. As secondary objectives, we aim to evaluate the transition readiness of CCSs, identify facilitating factors for transition and adherence to LTFU care, and compare the transition models of the three participating centers. The results of this study are intended to serve as a basis to improve transition and LTFU care in Switzerland and could be transferrable to countries with similar health care systems.

## Methods

### Study Design

The ACCS study is a prospective, multicenter, observational study and is registered at ClinicalTrials.gov (NCT04284189).

### Setting: Transition and LTFU Care Models in Participating Clinics

We included three pediatric oncology centers with different transition and LTFU care models—Center 1: Department of Pediatrics, Kantonsspital Luzern; Center 2: University Children’s Hospital Basel; and Center 3: Department of Pediatrics, Kantonsspital Aarau. All three centers are very similar in terms of the geographical catchment area, number of new diagnoses per year, and location of the adult hospital on the same campus. Center 1 refers most CCSs to the primary care physician but does not systematically make these referrals when CCSs reach a certain age or time since the completion of treatment. Some CCSs with already symptomatic late effects are transitioned to adult oncology. Center 2 transitions all CCSs to adult oncology and has done so since 2014. Their model includes one joint visit at the age of 18 years during which the adult oncologist or hematologist attends part of the pediatric clinic visit. The following visit takes place in the adult hospital with the adult oncologist or hematologist only. The transition model of Center 3 involves a transition process over a minimum of two clinical visits. The transition team, consisting of a designated pediatric oncologist and adult oncologist or hematologist, attends both appointments for the whole consultation time. The first visit takes place in the pediatric hospital, and the second visit takes place in the adult hospital. If the CCS feels comfortable, the third appointment is attended by the adult oncologist or hematologist only, but the pediatric oncologist is still available in case of specific questions.

### Eligibility Criteria and Group Assignment

CCSs are eligible to participate in the ACCS study if it has been at least 5 years since completion of their treatment, either first-line treatment or treatment for relapsed disease; if they were diagnosed with cancer according to the International Childhood Cancer Classification, third edition (ICCC3); if they were less than 18 years of age at cancer diagnosis; and if they are 16 years of age or older at the time of inclusion in the ACCS study. In addition, CCSs have to either be ready to transition from pediatric to adult-focused LTFU care (group 1) or already have been transitioned since 2014 (group 2). We chose the year 2014 because the standardized transition into adult oncology in Center 2 was established in that year. We exclude CCSs treated with surgery only and with no increased risk for late effects (eg, teratoma), those with ongoing cancer treatment or in a palliative situation, those with cognitive disabilities that would prevent the CCS from completing a questionnaire, and those not fluent in speaking and reading German.

### Recruitment Overview

We received approval from the cantonal ethics committee (Ethikkommission Nordwest- und Zentralschweiz) that is responsible for all three participating centers in February 2019 and started patient recruitment at all three study centers immediately following the approval. We plan to recruit participants over a period of 2 years, and we expect to have the results of the analyses of the questionnaires by autumn 2021.

The local investigator at each of the three sites is responsible for patient recruitment. After study initiation, each local investigator prepared a list with all eligible survivors for group 1 and group 2. During the recruitment period, each local investigator will include additional CCSs who meet the eligibility criteria.

### Recruitment and Study Time Points for Group 1 Childhood Cancer Survivors

**Figure 1:**
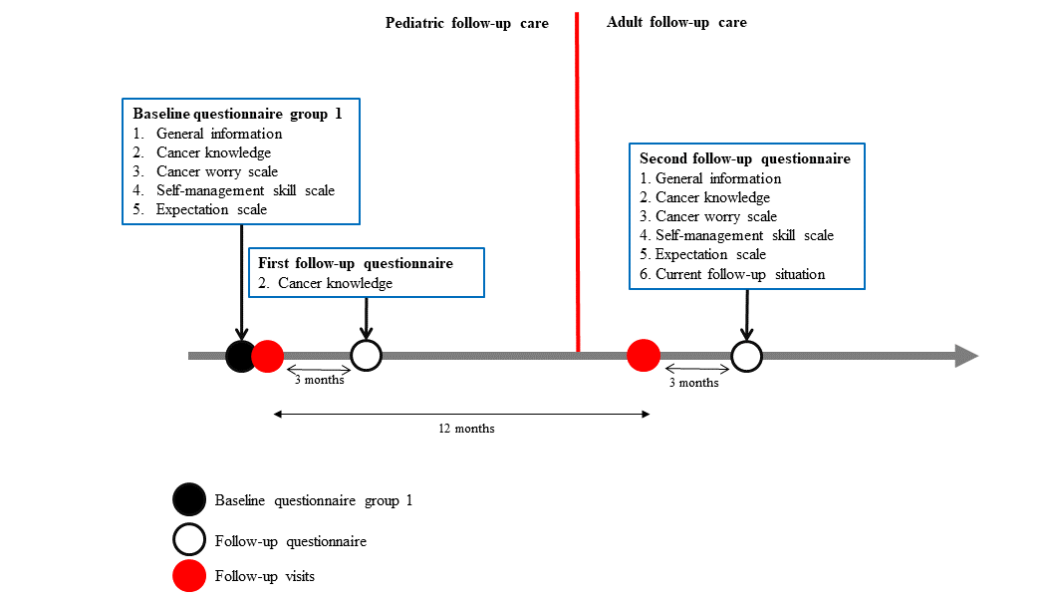
Timeline of the Aftercare of Childhood Cancer Survivors (ACCS) study and time points where group 1a participants answer questionnaires; questionnaire sections are numbered and identical numbers correspond to identical content.

**Figure 2:**
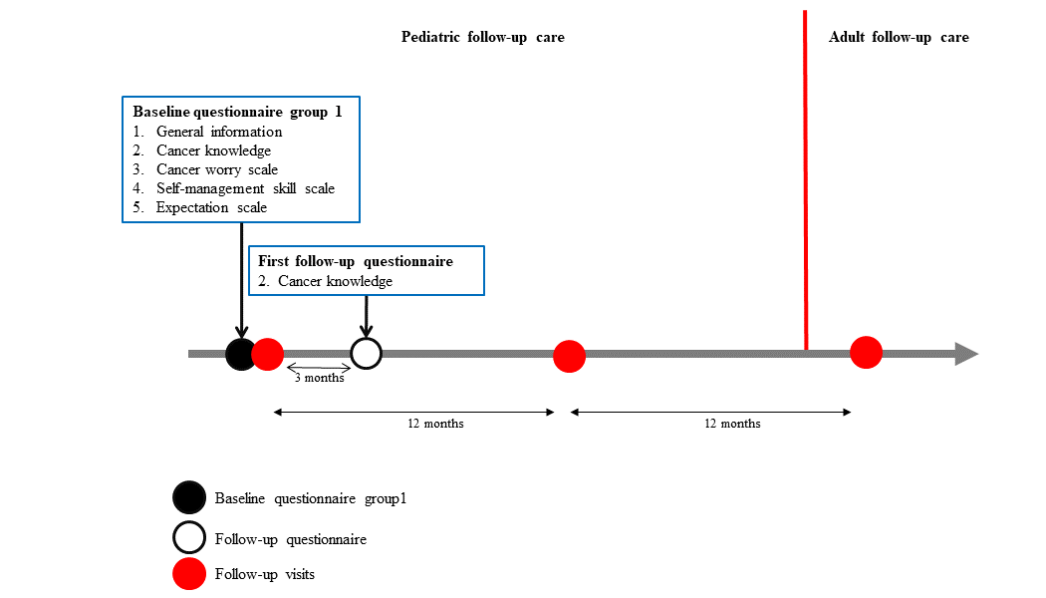
Timeline of the Aftercare of Childhood Cancer Survivors (ACCS) study and time points where group 1b participants answer questionnaires; questionnaire sections are numbered and identical numbers correspond to identical content.

Group 1 CCSs receive the study material by mail before their next scheduled visit at the pediatric LTFU clinic. The study material consists of an information letter, the informed consent form, the baseline questionnaire, a reply sheet to return if they do not want to participate, and a prepaid envelope to return the study documents before the next visit if they do want to participate. If an eligible CCS does not reply before the visit, the local investigator reminds him or her before the LTFU visit, and he or she still has the opportunity to participate. All group 1 CCSs receive the baseline questionnaire before the LTFU visit and the follow-up questionnaire 3 months after the LTFU visit. Group 1a consists of CCSs who decide to transition into adult care (see Figure 1). They receive a second follow-up questionnaire approximately 15 months after the first visit. An interval of 15 months was chosen because most survivors have roughly one LTFU visit per year. Therefore, with an interval of 15 months, the follow-up questionnaire is sent 3 months after the first visit in an adult setting. Whenever possible, we adapt the time at which the second follow-up questionnaire is sent according to the actual date of the follow-up visit in the adult setting. Group 1b consists of CCSs who decide during the LTFU visit to stay in pediatric follow-up care for another year (see Figure 2). They receive only the baseline questionnaire and first follow-up questionnaire after 3 months, which provide us with important information on transition readiness, related needs, and cancer knowledge.

The local investigators send one reminder only if the CCS does not respond within 4 weeks to the first and second follow-up questionnaires.

### Recruitment of Group 2 Childhood Cancer Survivors

**Figure 3:**
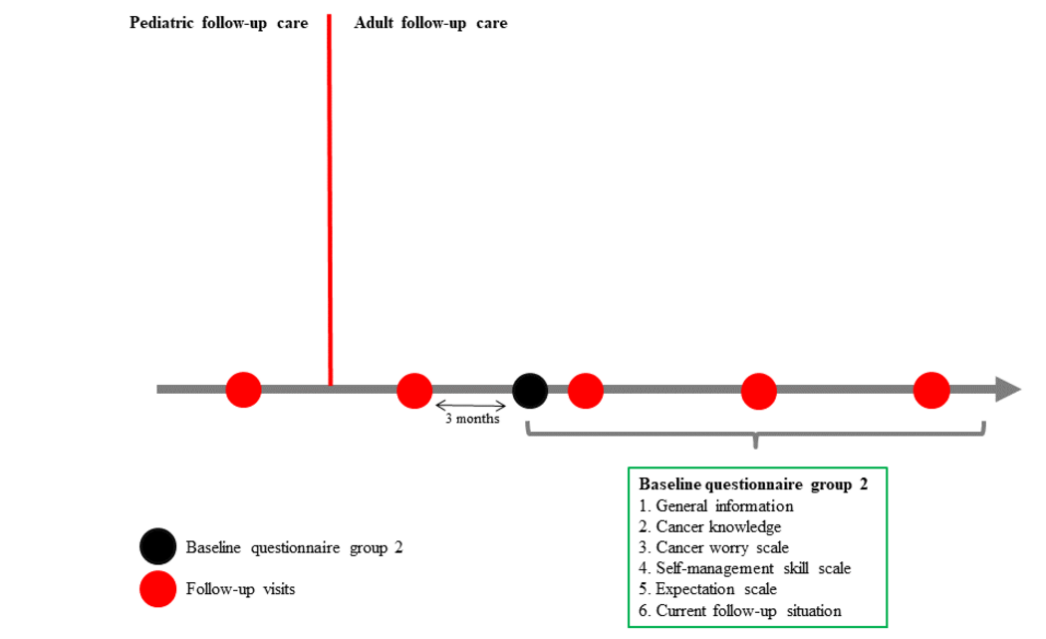
Timeline of the Aftercare of Childhood Cancer Survivors (ACCS) study and time point where group 2 participants answer the questionnaire.

The local investigators contact group 2 CCSs who have already left pediatric follow-up care at the start of the study. The time since transition might range from 3 months to 6 years (see Figure 3). These CCSs receive the study material by mail and a maximum of one reminder if they do not reply within 4 weeks. Group 2 CCSs receive only one baseline questionnaire. The information provided by group 2 CCSs is crucial to assess cancer knowledge and needs related to transition and LTFU care in adult CCSs. In addition, comparing the answers of former and currently transitioned CCSs will enable us to evaluate whether transition practices in each participating center improved over time from the CCS point of view.

### Data Collection and Questionnaire Content

#### Baseline Questionnaires for Childhood Cancer Survivors

All participants receive a baseline questionnaire, which is largely identical for survivors in group 1 and group 2: Baseline questionnaire group 1 and Baseline questionnaire group 2. The baseline questionnaires provide us with information on CCSs’ cancer knowledge, current follow-up situation, and needs. In both groups, we collect data on CSSs’ current age, sex, highest completed or ongoing education level, and the subjective assessment of current health status. We assess CCSs’ knowledge on their cancer diagnosis, treatment modalities received, and potential late effects using questions with the answer options “yes,” “no,” and “unsure.” Subsequently, we assess cancer worry, self-management skills, and expectations for follow-up care using validated scales. The Cancer Worry Scale consists of six questions on how much CCSs worry about their cancer history, relapse, fertility, late effects, and secondary malignancy [18]. The Self-Management Skill Scale consists of 15 questions, which cover factors important for evaluating CCSs’ independence and personal responsibility [18]. The 12 questions of the Expectations Scale cover a broad spectrum of expectations concerning the treatment team and organizational or structural processes in the clinics [18]. For all three scales, the questions use Likert response scales with the following options: “strongly disagree,” “disagree,” “agree,” and “strongly agree.”

The baseline questionnaire of group 2 CCSs contains one additional question that the group 1 baseline questionnaire does not contain, which asks about the current follow-up situation. The response indicates whether the CCS is continuing with follow-up care and either where the follow-up care takes place or why the CCS discontinued follow-up care.

#### Follow-Up Questionnaires for Childhood Cancer Survivors

The first follow-up questionnaire for group 1 CCSs, including groups 1a and 1b, only asks about cancer knowledge. The content of the second follow-up questionnaire is identical to that of the baseline questionnaire of group 2 CCSs.

#### Collection of Medical Data

We collect medical data using a questionnaire completed by the local investigators. The local investigators answer five questions regarding tumor diagnosis: main category of cancer diagnosis (eg, leukemia), diagnosis according to the ICCC3, tumor location, whether the patient has suffered from a relapse, and patient’s age at diagnosis. In addition, the local investigators indicate CCSs’ treatment exposure and organ-specific risks for late effects. The local investigators extract these data from medical records.

### Data Management

For electronic acquisition of the paper-based questionnaires, we use the software Remark Office OMR (Gravic, Inc). After each questionnaire version has been edited with the software, the software recognizes the selected answers on the completed documents and saves the answers electronically. The software traces changes in the database via an audit trail. The data stored in Remark Office OMR can be exported as Excel documents, which allows further processing of the data by statistical software, such as Stata (StataCorp LLC) and R (The R Foundation).

### Statistical Analysis

The ACCS study has two analytical approaches: a cross-sectional approach and a longitudinal approach. The cross-sectional approach includes analysis of the baseline questionnaire from group 2 CCSs and the second follow-up questionnaire from group 1 CCSs. Both questionnaires contain the same questions and answer options, with the exception of the “Current follow-up situation” question, which is asked only for group 2 CCSs. For the cross-sectional approach, we will present the results mainly descriptively (ie, mean and median with an appropriate measure of spread as well as summary tables and graphs), for example, the distribution of the four answer options for the questions on cancer worries. Additionally, we will quantify the associations between survivors’ worries or expectations regarding follow-up care and diagnosis, treatment exposure, or the risk of developing late effects based on specific parameters such as odds ratio with respective measures of distribution.

The longitudinal approach includes the analysis of questionnaires from group 1 CCSs who answer at least one follow-up questionnaire. The answers from the group 1a and 1b CCSs can be used to determine whether follow-up consultation improves cancer knowledge. For the analysis of CCSs’ satisfaction and expectations over time, we will use data from group 1b CCSs who answer the baseline and second follow-up questionnaire. In addition to using descriptive analysis as in the cross-sectional approach, we will use analysis of covariance in the longitudinal approach, whereby the result of the baseline questionnaire will serve as a covariate in the model for the result of the second follow-up questionnaire. In addition, we will analyze and discuss the different transition models taking into account the follow-up situation of each center when the project started. We will use the statistical software programs Stata and R.

## Results

To date, we have recruited 58 CCSs in total, with 33 participants from the Division of Oncology-Hematology at Kantonsspital Aarau. We use this center to provide a practical illustration of the recruitment process and the current status. The center invited 72 CCSs to participate in the study in the first year. A total of 33 CCSs (46%) have confirmed their participation and completed the baseline questionnaire. A total of 19 of these CCSs are in group 1 and have already completed the first follow-up questionnaire, and 14 CCSs belong to group 2. Of the remaining 39 CCSs, 10 were recently contacted for the first time, whose responses are still pending, and 16 did not respond; of these 16 nonresponders, 5 stated no interest in the study, 2 could not be included due to medical reasons, 2 did not show up for the visit, and 1 stated insufficient time for participation. The recruitment will continue until January 2021.

## Discussion

### Overview

In this study, we will assess the follow-up situation, related needs, and knowledge of AYA cancer survivors who transition from pediatric to adult-focused follow-up care. We will evaluate their transition readiness, identify facilitators for transition and adherence to LTFU care, and compare the transition models of the three participating centers.

### Strength and Limitations

This is the first study in Switzerland assessing CCSs’ needs and perceptions of transition and LTFU care using a prospective, multicenter, observational approach. The survivors’ feedback on the study has been mostly positive. At this preliminary state, we assume that the questionnaires are understandable and easy to complete, as most participants have completed all questions, and no questions regarding content have arisen during the clinic visits. Remark Office OMR is an easy-to-use tool for scanning and analyzing questionnaires, as it eliminates the need to manually transcribe the original questionnaires.

The questionnaire-based design might introduce selection bias, as CCSs who are interested in LTFU care are potentially more willing to participate than those who are not interested. For CCSs who are still in follow-up care, social desirability bias might positively influence their answers and prevent them from being critical. The interval between the last LTFU care visit to the completion of the questionnaire among the group 2 CCSs is variable and ranges theoretically from 3 months to 6 years (inclusion starts from 2014 onward).

### Lessons Learned

Setting up and participating in a prospective study requires the willingness of all local investigators to continuously work on the project. For the ACCS study, this means always thinking about CCSs who are potentially eligible to participate in the study and sending them the documents, reminders, and follow-up questionnaires. Close collaboration and site visits by the primary investigator are essential. Regarding patient recruitment, we have learned that some CCSs prefer oral information to written information. We noticed this during the clinical visits when we asked survivors eligible for group 1 who had not returned the documents beforehand why they had not returned them. By providing oral information, we could recruit more participants. It is, therefore, worthwhile to talk to the survivors personally about the planned study.

### Conclusions

The ACCS study collects detailed information on CCSs’ preferences and expectations regarding LTFU care and the transition into the adult setting. We need these results to adapt LTFU care to CCSs’ needs and to identify areas where targeted interventions are possible, such as patient education. Through this approach and a subsequent well-structured, standardized transition focused on the survivor’s needs, adherence to LTFU care can be improved and loss to follow-up can be reduced.

## Abbreviations

ACCS: Aftercare of Childhood Cancer Survivors
AYA: adolescent and young adult
CCS: childhood cancer survivor
ICCC3: International Childhood Cancer Classification, third edition
LTFU: long-term follow-up
